# Genome Sequence of *Euphorbia mosaic virus* from Passionfruit and *Euphorbia heterophylla* in Florida

**DOI:** 10.1128/genomeA.01714-16

**Published:** 2017-03-02

**Authors:** J. E. Polston, M. A. Londoño, A. L. Cohen, M. Padilla-Rodriguez, K. Rosario, M. Breitbart

**Affiliations:** a Department of Plant Pathology, University of Florida, Gainesville, Florida, USA; b College of Marine Science, University of South Florida, St. Petersburg, Florida, USA

## Abstract

*Euphorbia mosaic virus* (EuMV) was found in a symptomatic passionfruit (*Passiflora edulis*) plant from Homestead, Florida, USA, as well as in the symptomatic weed *Euphorbia heterophylla*. This is the first identification of EuMV in Florida and the United States and the first report of a natural infection of passionfruit by EuMV.

## GENOME ANNOUNCEMENT

*Euphorbia mosaic virus* (EuMV) is a species of *Begomovirus* (*Geminiviridae*), a taxon of plant viruses characterized by single-stranded circular DNA genomes (1). EuMV has a bipartite genome consisting of a DNA-A (2,609 to 2,615 nucleotides [nt]) and a DNA-B (2,571 to 2,590 nt).

DNA was extracted from the leaves of a passionfruit plant (*Passiflora* *edulis* Sims) showing symptoms of leave distortion and necrotic spots (2). The leaves were collected in 1993 from Homestead, Florida, USA, desiccated, and stored at 4°C. A DNA-A (2,609 nt; KJ647290), and a DNA-B (2,545 nt; KJ647291) were cloned from *EcoR*I and *Ava*I-digestion of rolling-circle amplification products generated using random hexamers. Pairwise scores generated by the Species Demarcation Tool (SDT) indicated that the DNA-A and the DNA-B had the greatest similarity, 98.6% and 97.2%, respectively, to the DNA-A and the DNA-B of EuMV-[CU:Hav:27:07] obtained from *Euphorbia* *heterophylla* L. in Cuba (HQ896201, HQ896201) (3, 4). The common regions of the DNA components (344 nt) were 97% identical, indicating that these constitute an isolate of *Euphorbia mosaic virus* (EuMV-[US:Fl:PF:313:1993]).

Samples of *E. heterophylla* plants showing symptoms of bright foliar mosaic were collected from Homestead, Florida, in 2013. A DNA-A (2,609 nt; JQ963887) and a DNA-B (2,585 nt; JQ963888) were obtained through cloning and sequencing of *Xmn*I-digested rolling-circle amplification products generated using random hexamers. SDT pairwise scores indicated that the DNA-A and the DNA-B had their highest identities, 98.8% and 98.1%, respectively, with those of EuMV-[US:Fl:PF:313:1993]. The common regions of the components (341 nt) were 97% identical, indicating that these constitute a bipartite begomovirus, designated EuMV-[US:Fl:Eu4]. The B component sequences differed in size due to a 40-nt deletion near the iterons in EuMV-[US:Fl:PF:313:1993]. While the presence of a begomovirus in symptomatic *E. heterophylla* has been known in Homestead, Florida, for many years (5), EuMV is the first virus to be associated with those disease symptoms.

DNA-A and DNA-B clones of EuMV-[US:Fl:PF:313:1993] were successfully inoculated to passionfruit ‘Lilikoi’ and *Phaseolus vulgaris* ‘Topcrop’ (6). Symptoms in *P. edulis* began as a mild mottling followed by necrotic spots, leaf deformation, and flower abortion. These symptoms are similar to those described for *Passionfruit severe leaf distortion virus* (from Brazil) but different from those of two other partially characterized begomoviruses (7–9). Symptoms in *P. vulgaris*, severe leaf distortion and stunting, were similar to those described for EuMV-YP from Mexico (10). Whitefly adults (*Bemisia* *tabaci* Genn. MEAM1) successfully transmitted EuMV from bean to bean, but not from passionfruit to either bean or passionfruit (11). *P. edulis* has been reported to be a poor colonization host for the MEAM1 whitefly (12).

Surveys of passionfruit in Homestead, Florida, from 2011 to 2012 failed to identify any EuMV-infected passionfruit plants, although EuMV-infected *E. heterophylla* plants with whiteflies were readily found. EuMV-[US:Fl:PF:313:1993] may have been transmitted from *E. heterophylla* to passionfruit by a whitefly that was later displaced by *B. tabaci* MEAM1, which first appeared in Florida in the mid-1980s (13, 14).

To our knowledge, this is the first report of EuMV as the causal agent of a disease in passionfruit and the first report of EuMV in the United States.

### Accession number(s).

The sequences of EuMV-[US:Fl:PF:313:1993] were deposited in GenBank under the accession numbers KJ647290 and KJ647291 (DNA-A and DNA-B, respectively), and the sequences of EUMV-[US:Fl:Eu4] were deposited in GenBank under the accession numbers JQ963887 and JQ963888.
